# Supplementary material for: Frameshift variant in MITF gene in a large family with Waardenburg syndrome type II and a co-segregation of a C2orf74 variant
Source: PLoS One. 2021 Feb 11;16(2):e0246607. doi: 10.1371/journal.pone.0246607 (PMC7877624; doi:10.1371/journal.pone.0246607)
Supplement: Attachment — Submitted filename: Response to Reviewers.docx [file pone.0246607.s002.docx]

PONE-D-20-28113
Frameshift variant in MITF gene in a large family with Waardenburg Syndrome type II and a Co-segregation of a C2orf74 variant
PLOS ONE

Dear Editor

I am very thankful to you for sending the manuscript to reviewers for evaluation. We have revised the manuscript in view of reviewer’s comments/suggestions. Moreover, manuscript has been formatted according to the PLOS ONE style. Furthermore, raw data has been deposited to the public repositories. Changes made in the manuscript has been highlighted. Please find below point-by-point response to the comments.

 Editorial Comments:

Comment

1. Please ensure that your manuscript meets PLOS ONE's style requirements, including those for file naming. The PLOS ONE style templates can be found at

<https://journals.plos.org/plosone/s/file?id=wjVg/PLOSOne_formatting_sample_main_body.pdf> and

<https://journals.plos.org/plosone/s/file?id=ba62/PLOSOne_formatting_sample_title_authors_affiliations.pdf>

Response:

Manuscript has been formatted according to the PLOS ONE style.

Comment

2. We note that you have indicated that data from this study are available upon request. PLOS only allows data to be available upon request if there are legal or ethical restrictions on sharing data publicly. For more information on unacceptable data access restrictions, please see <http://journals.plos.org/plosone/s/data-availability#loc-unacceptable-data-access-restrictions>.

In your revised cover letter, please address the following prompts:

 a) If there are ethical or legal restrictions on sharing a de-identified data set, please explain them in detail (e.g., data contain potentially sensitive information, data are owned by a third-party organization, etc.) and who has imposed them (e.g., an ethics committee). Please also provide contact information for a data access committee, ethics committee, or other institutional body to which data requests may be sent.

b) If there are no restrictions, please upload the minimal anonymized data set necessary to replicate your study findings as either Supporting Information files or to a stable, public repository and provide us with the relevant URLs, DOIs, or accession numbers. For a list of acceptable repositories, please see <http://journals.plos.org/plosone/s/data-availability#loc-recommended-repositories>.

We will update your Data Availability statement on your behalf to reflect the information you provide.

Response:

Raw reads (fastq files) have been uploaded to sequence read archives
(SRA) of NCBI. The project details can be accessed using the BioProject accession number PRJNA687138. Link for data accession is <https://www.ncbi.nlm.nih.gov/Traces/study/?acc=PRJNA687138>

Comment

3. Your ethics statement should only appear in the Methods section of your manuscript. If your ethics statement is written in any section besides the Methods, please delete it from any other section.

Response:

Moved to Methods section

Reviewers' comments:

Reviewer's Responses to Questions

**Comments to the Author**

1. Is the manuscript technically sound, and do the data support the conclusions?

The manuscript must describe a technically sound piece of scientific research with data that supports the conclusions. Experiments must have been conducted rigorously, with appropriate controls, replication, and sample sizes. The conclusions must be drawn appropriately based on the data presented.

Reviewer #1: Yes

Reviewer #2: Partly

2. Has the statistical analysis been performed appropriately and rigorously?

Reviewer #1: Yes

Reviewer #2: Yes

3. Have the authors made all data underlying the findings in their manuscript fully available?

The [PLOS Data policy](http://www.plosone.org/static/policies.action#sharing) requires authors to make all data underlying the findings described in their manuscript fully available without restriction, with rare exception (please refer to the Data Availability Statement in the manuscript PDF file). The data should be provided as part of the manuscript or its supporting information, or deposited to a public repository. For example, in addition to summary statistics, the data points behind means, medians and variance measures should be available. If there are restrictions on publicly sharing data—e.g. participant privacy or use of data from a third party—those must be specified.

Reviewer #1: No

Reviewer #2: No

4. Is the manuscript presented in an intelligible fashion and written in standard English?

PLOS ONE does not copyedit accepted manuscripts, so the language in submitted articles must be clear, correct, and unambiguous. Any typographical or grammatical errors should be corrected at revision, so please note any specific errors here.

Reviewer #1: Yes

Reviewer #2: Yes

5. Review Comments to the Author

Please use the space provided to explain your answers to the questions above. You may also include additional comments for the author, including concerns about dual publication, research ethics, or publication ethics. (Please upload your review as an attachment if it exceeds 20,000 characters)

Comment

Reviewer #1: I have no major concern. I only have 2 minor issues-
1) In the whole exome sequencing studies, please outline the filtering steps undertaken to reach at 67 coding variants.
Response:

Following statement has been added to the text. Please see page 6.

Various filter were applied to search for potential candidate variants including depth of coverage (DP >10), genotype quality (GQ >20), and unreported SNP. Only variants with low allelic frequency (below 1 % in 1000G, ExAC) were taken into consideration. These settings yielded 67 rare coding variants.

Comment

2) please make all data publicly available and not by request to author. Please upload mutation data to dbSNP.

Response:

Raw reads (fastq files) have been uploaded to sequence read archives
(SRA) of NCBI. The project details can be accessed using the BioProject accession number PRJNA687138. Link for data accession is <https://www.ncbi.nlm.nih.gov/Traces/study/?acc=PRJNA687138>

Reviewer #2: In this manuscript, the authors found that the mutations in the MITF gene and C2orf74 are potentially the causal factor for a family with multiple individuals affected by Waardenburg syndrome type II. The potential mutants were first identified in two affected individuals with whole-exome sequencing (WES) and then verified in other members with Sanger sequencing. Based on the genotypes of family members, MITF and C2orf74 are highly likely the genes leading to this disease in this family.

Comment: My first concern is that not enough details were provided in the genetic analysis. More details need to be provided instead of simply saying “Various filters were applied and coding variants were reduced to 67”. Otherwise, the chosen mutation can be an artifact that happens to segregate well. Additionally, the WES data or genotype data is not available for validation by other researchers. Finally, there are quite many grammar errors that need to be fixed (see below for some examples).
Response: Various filter were applied to search for potential candidate variants including depth of coverage (DP >10), genotype quality (GQ >20), and unreported SNP. Only variants with low allelic frequency (below 1 % in 1000G, ExAC) were taken into consideration. These settings yielded 67 rare coding variants.

Comment: Page 9: change “Further subtypes do exists” to “Further subtypes do exist”.

Response: Fixed

Comment: Page 9: change “an individual patients” to “an individual patient”.

Response: Fixed

Comment: Page 9: change “mutations in PAX3 gene is” to “mutation of PAX3 gene is”.

Response: Done

Comment: Page 9: change “Majority of” to “The majority of”.

Response: Done

Comment: Page 10: change “20-30% patients” to “20-30% of patients”.

Response: Done

Comment: Page 11: change “The read length of the paired end” to “The length of the paired end read”.

Response: Done

Comment: Page 11: “Reads were aligned to the human reference genome (GRCh38/hg38) and variants were annotated”. How variants were called?

Response: Genome Analysis Toolkit (GATK) was used to call the variants.

Comment: Page 11: change “variant specific” to “variant-specific”.

Response: Done

Comment: Page 11: change “Detailed examination rule out presence” to “Detailed examination ruled out the presence”

Response: Done

Comment: Page 12: change “hypothesis free” to “hypothesis-free”.

Response: Done

Comment: Page 12: change “the variants in MITF and C2orf74 segregates” to “the variants in MITF and C2orf74 segregate”.

Response: Done

Comment: Page 12: change “Sanger sequencing validate” to “Sanger sequencing validates”.

Response: Done

Comment: Page 13: change “It is highly heterogeneous syndrome” to “It is a highly heterogeneous syndrome”

Response: Done

Comment: Page 14: references were needed for “Mutations in melanocyte inducing transcription factor (MITF), coding for a basic helix-loop-helix (BHLH) leucine zipper protein, are known to cause the WS2 phenotype”.

Response: References added

6. PLOS authors have the option to publish the peer review history of their article ([what does this mean?](https://journals.plos.org/plosone/s/editorial-and-peer-review-process#loc-peer-review-history)). If published, this will include your full peer review and any attached files.


If you choose “no”, your identity will remain anonymous but your review may still be made public.


**Do you want your identity to be public for this peer review?** For information about this choice, including consent withdrawal, please see our [Privacy Policy](https://www.plos.org/privacy-policy).

Reviewer #1: No

Reviewer #2: No

[NOTE: If reviewer comments were submitted as an attachment file, they will be attached to this email and accessible via the submission site. Please log into your account, locate the manuscript record, and check for the action link "View Attachments". If this link does not appear, there are no attachment files.]

While revising your submission, please upload your figure files to the Preflight Analysis and Conversion Engine (PACE) digital diagnostic tool, <https://pacev2.apexcovantage.com/>. PACE helps ensure that figures meet PLOS requirements. To use PACE, you must first register as a user. Registration is free. Then, login and navigate to the UPLOAD tab, where you will find detailed instructions on how to use the tool. If you encounter any issues or have any questions when using PACE, please email PLOS at [figures@plos.org](mailto:figures@plos.org). Please note that Supporting Information files do not need this step.
