# Supplementary material for: Frameshift variant in MITF gene in a large family with Waardenburg syndrome type II and a co-segregation of a C2orf74 variant
Source: PLoS One. 2021 Feb 11;16(2):e0246607. doi: 10.1371/journal.pone.0246607 (PMC7877624; doi:10.1371/journal.pone.0246607)
Supplement: Attachment — Submitted filename: Response to Reviewer comments.docx [file pone.0246607.s003.docx]

PONE-D-20-28113R1
Frameshift variant in MITF gene in a large family with Waardenburg Syndrome type II and a Co-segregation of a C2orf74 variant


Dear Editor

I am very thankful to you for providing me the comments of a reviewer on the revised version of the manuscript. We have revised the manuscript in view of reviewer’s comments/suggestions. Please find below response to the comments of a reviewer.

Reviewer #1: (No Response)

Reviewer #2: The authors have addressed my concerns. Just some suggestions: add software version for GATK and others. For gnomAD and other databases, the version is also necessary. Also, it will be recommended to include a table to summarize the 67 variants (or just some more likely variants), including their location in genome, allele frequency in gnomAD, gene, protein change, mutation affect prediction, number of individuals with the variant, and other important information that showing that they are risk mutations.

Response

Version numbers for GATK, dbSNP, and GnomAD have been added. Moreover a table containing information of all 67 variants have been added as a supplementary data. Please see supplementary table 1.
